# Increased BMP/SMAD Signaling by PD-MSCs Promotes Bone Formation in an Ovariectomized Mouse Model of Osteoporosis

**DOI:** 10.3390/ijms262010017

**Published:** 2025-10-15

**Authors:** Dae Hyun Lee, Hyeri Park, Sihyun Kim, Jong Ho Choi, Sang Shin Lee, Gi Jin Kim

**Affiliations:** 1 Department of Convergence Science, CHA University, Seongnam 13488, Republic of Korea; ldh1532@chauniv.ac.kr (D.H.L.); hyeyeyeri@gmail.com (H.P.); 2 Department of Oral Pathology, Gangneung-Wonju National University, Gangneung 25457, Republic of Korea; shihyun@hanmail.net (S.K.); jhchoi@gwnu.ac.kr (J.H.C.); sangshin@gwnu.ac.kr (S.S.L.)

**Keywords:** mesenchymal stem cell, osteoporosis, ovariectomy, osteoblast, osteoclast, bone morohgenic protein (BMP), SMAD

## Abstract

Mesenchymal stem cells (MSCs) have emerged as a promising therapeutic approach for degenerative diseases due to their ability to modulate disease progression through paracrine mechanisms. Among various MSC sources, placenta-derived MSCs (PD-MSCs) offer significant advantages, including high proliferation capacity, reduced senescence, and low immunogenicity, making them ideal for allogeneic applications. In this study, we investigated the therapeutic effects of PD-MSC transplantation in an estrogen-deficiency-induced osteoporosis mouse model. Mice were divided into three groups: a normal control group, a non-transplanted osteoporosis group, and a PD-MSC-transplanted group. Our findings demonstrated that PD-MSC transplantation significantly improved osteoporosis-related parameters, including increased femur weight, bone volume, bone mineral density, and calcium deposition. Additionally, estrogen levels were elevated, bone formation markers were upregulated, and bone resorption markers were downregulated. PD-MSCs also reduced inflammatory cytokine levels while enhancing anti-inflammatory factors. Notably, the BMP/SMAD signaling pathway, crucial for bone formation, was significantly upregulated. These results suggest that PD-MSC transplantation effectively restores bone homeostasis by inhibiting osteoclast activity, promoting osteogenesis, and modulating inflammation. This study provides strong evidence supporting the potential of PD-MSCs as a novel therapeutic strategy for osteoporosis, offering a regenerative and anti-inflammatory approach to bone disease management.

## 1. Introduction

Osteoporosis is a bone disease where bones become porous, leading to weakened bone strength and a higher risk of fractures. It is a major global health issue, affecting over 200 million people and causing more than 8.9 million fractures each year, mostly in the hip. The impact is significant, with costs around $17 billion in the U.S. alone [1].

Preventing osteoporosis and fractures is vital for maintaining health and independence, especially among the elderly. In women, osteoporosis is often linked to menopause, which decreases estrogen levels and accelerates bone loss [2]. Estrogen helps regulate bone remodeling, so its deficiency plays a key role in postmenopausal osteoporosis [3]. Current treatments for postmenopausal osteoporosis include medications such as bisphosphonates and lifestyle modifications including calcium and vitamin D supplementation. While these remain the cornerstone of fracture prevention, recent studies have redefined their pharmacologic roles. Bisphosphonates remain indispensable due to their sustained antiresorptive activity and ability to consolidate the effects of sequential therapies even after discontinuation [4]. Vitamin D, beyond its classical role in calcium–phosphorus homeostasis, also modulates immune and inflammatory pathways, further influencing bone metabolism [5]. In addition, natural bioactive compounds such as flavonoids have emerged as promising candidates with dual osteogenic and antiresorptive mechanisms, offering innovative perspectives for future osteoporosis management [6]. There is a growing need for new medications with innovative mechanisms to address these issues and provide better options for managing osteoporosis [7]. The pursuit of innovative therapeutic options aims to offer effective alternatives that can overcome the limitations and challenges posed by traditional osteoporosis treatments.

Maintaining bone homeostasis involves a dynamic and continuous process of bone remodeling, orchestrated by three primary cell types: osteoblasts, osteoclasts, and osteocytes. Osteoclasts are responsible for the resorption of old bones, while osteoblasts are involved in the formation of new bones. This process allows for the repair of micro-injuries and adaptation to mechanical stress. After the bone matrix is mineralized, osteoblasts mature into osteocytes, which act as mechanosensory and regulate the remodeling process [8]. This intricate balance is influenced by a variety of local and systemic factors, including growth factors, cytokines, chemokines, and hormones such as estrogen [9,10]. Under normal conditions, the activities of osteoclasts and osteoblasts are tightly coupled, resulting in the complete replacement of resorbed bone with newly formed bone. Disruption of this equilibrium—such as through excessive osteoclast activity—can lead to bone pathologies like osteoporosis, Paget’s disease, rheumatoid arthritis, and osteoarthritis [11]. Conversely, impaired osteoclast function or abnormal osteoblast activity can result in conditions such as osteopetrosis, osteomalacia, or rickets. A thorough understanding of these mechanisms is crucial for elucidating disease processes and developing effective therapies for bone-related disorders.

The potential applications of mesenchymal stromal cells (MSCs) sourced from diverse origins such as bone marrow (BM), adipose tissue, and the human term placenta have been suggested for a range of clinical purposes. A growing body of evidence indicates that the effectiveness of various MSCs lies in their indirect actions, primarily through the paracrine release of pro-regenerative, anti-inflammatory, and antioxidant factors [12]. Notably, utilizing BM-derived MSCs for autologous applications is hindered by the limited yield of cells obtained and the invasiveness of the extraction procedure. Comparable therapeutic effects have been observed with both autologous and allogeneic MSCs isolated from different tissues [13]. The readily available and rich source of human MSCs in fresh placental tissue appears to be particularly advantageous for harvesting abundant cells suitable for allogenic use. Cells obtained from neonatal tissues of the placenta exhibit the remarkable capability of prolonged expansion with a low susceptibility to senescence. Placenta-derived MSCs (PD-MSCs) present a distinct advantage in allogeneic or xenogeneic applications in normal immunocompetent hosts due to their low expression of HLA class I and II, thereby minimizing the risk of rejection [14]. The primary strength of therapies based on PD-MSCs lies in their secretion of a diverse array of pro-regenerative, anti-inflammatory, and antioxidant factors. This unique feature positions PD-MSCs as highly competent cells for therapeutic interventions, whether in the context of human applications or in animal models. Previous research has demonstrated that in vivo transplantation of mesenchymal stem cells (MSCs) can repair osteoporotic bone damage [15,16].

The potential applications of mesenchymal stromal cells (MSCs) sourced from diverse origins such as bone marrow (BM), adipose tissue, and the human term placenta have been suggested for a range of clinical purposes. A growing body of evidence indicates that the effectiveness of various MSCs lies in their indirect actions, primarily through the paracrine release of pro-regenerative, anti-inflammatory, and antioxidant factors [12]. Notably, utilizing BM-derived MSCs for autologous applications is hindered by the limited yield of cells obtained and the invasiveness of the extraction procedure. Comparable therapeutic effects have been observed with both autologous and allogeneic MSCs isolated from different tissues [13]. The readily available and rich source of human MSCs in fresh placental tissue appears to be particularly advantageous for harvesting abundant cells suitable for allogenic use. Cells obtained from neonatal tissues of the placenta exhibit the remarkable capability of prolonged expansion with a low susceptibility to senescence. Placenta-derived MSCs (PD-MSCs) present a distinct advantage in allogeneic or xenogeneic applications in normal immunocompetent hosts due to their low expression of HLA class I and II, thereby minimizing the risk of rejection [14]. The primary strength of therapies based on PD-MSCs lies in their secretion of a diverse array of pro-regenerative, anti-inflammatory, and antioxidant factors. This unique feature positions PD-MSCs as highly competent cells for therapeutic interventions, whether in the context of human applications or in animal models. Previous research has demonstrated that in vivo transplantation of mesenchymal stem cells (MSCs) can repair osteoporotic bone damage [15,16].

To investigate the potential impact of PD-MSCs transplantation as a novel treatment for osteoporosis, an ovariectomy (OVX) approach was employed in mice [17,18]. OVX involves the removal of ovaries, the primary source of estrogen, making it a widely utilized and excellent preclinical model for si\4-mulating postmenopausal osteoporosis. This model effectively mimics estrogen deficiency-induced bone loss and exhibits clinical manifestations akin to postmenopausal osteoporosis. The ovariectomized animal model of osteoporosis serves as a valuable tool for studying the efficacy of various therapeutic agents, including established treatments like estrogen and bisphosphonates [19]. This approach allows researchers to assess the potential of novel treatments, such as PD-MSCs transplantation, in addressing the specific challenges and characteristics associated with osteoporosis induced by estrogen deficiency.

## 2. Results

### 2.1. Characterization of PD-MSCs

**Figure 1:**
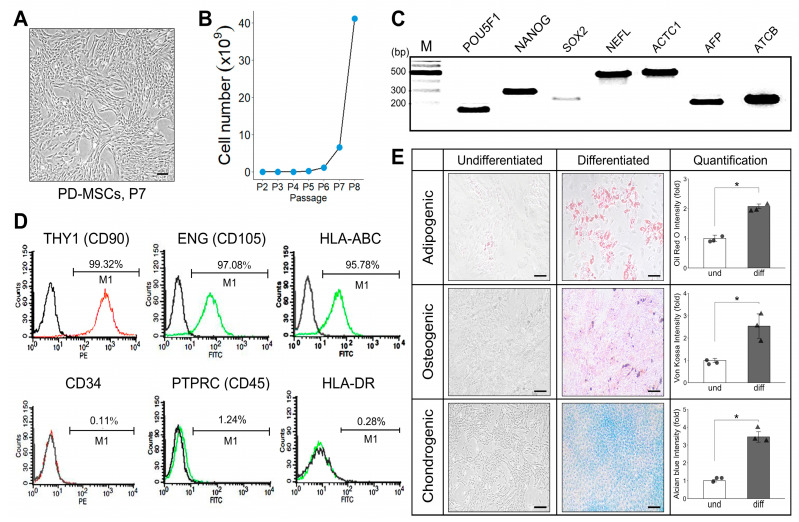
Characterization of PD-MSCs. (**A**) The morphology of PD-MSCs. (**B**) The cell number accumulation during the proliferation of PD-MSCs (**C**) mRNA expression of Stemness markers analyzed by RT-PCR. (**D**) Expression of surface markers for immunomodulation in PD-MSCs. (**E**) Adipogenic, osteogenic and chondrogenic differentiation were confirmed by Oil-Red O staining, von Kossa staining, Alcian Blue staining. Scale bar: (**A**) 200 μm, (**E**) 100 μm. Data are shown as mean ± SD. The Mann–Whitney U test was used to compare two independent groups, suitable for non-normally distributed data. A *p*-value < 0.05 was considered statistically significant, * *p* < 0.05.

To confirm the PD-MSC characteristics, we analyzed the morphology and proliferation capacity of PD-MSCs. PD-MSCs exhibited an elongated, spindle-shaped morphology and kept growing after passaging (Figure 1A,B). The analysis of stemness markers and the expressions of three germ lineages of PD-MSCs was performed using end-point RT-PCR. These cells expressed self-renewal markers such as POU5F1 (Oct4), NANOG, and SOX2, indicating their capacity for self-regeneration. Furthermore, germ lineage-specific markers, including NEFL, ACTC1, and AFP, were expressed, confirming the characterization of these MSCs (Figure 1C). Isolated PD-MSCs displayed typical MSC phenotypes, expressing markers such as THY1 (CD90), ENG (CD105), HLA-ABC, and lacking CD34, PTPRC (CD45), HLA-DR (Figure 1D). The differentiation capabilities of PD-MSCs across adipogenic, osteogenic, and chondrogenic lineages were confirmed using specific staining methods, including Oil-Red O for adipogenesis, von Kossa staining for osteogenesis, and alcian blue staining for chondrogenesis (Figure 1E).

### 2.2. PD-MSC Engraftment Restores Osteoporotic Femoral Structure in OVX Mice

To investigate the therapeutic potential of human PD-MSCs, in an osteoporosis model, mice underwent OVX following the protocol outlined in the methods section. In brief, OVX was performed on 7-week-old female C57BL/6 mice through two dorsolateral skin incisions, involving the surgical removal of ovaries with a sterile suture while under anesthesia with avertin. A total of mice was used, with randomly assigned to each of the three groups: the normal group (Nor), non-transplantation (NTx) group, and transplantation (Tx) group. Four weeks post-OVX, PD-MSCs (5 × 10^4^ cells), obtained from the human placental chorionic plate and cultured, were intravenously injected into the Tx group through the tail vein.

**Figure 2:**
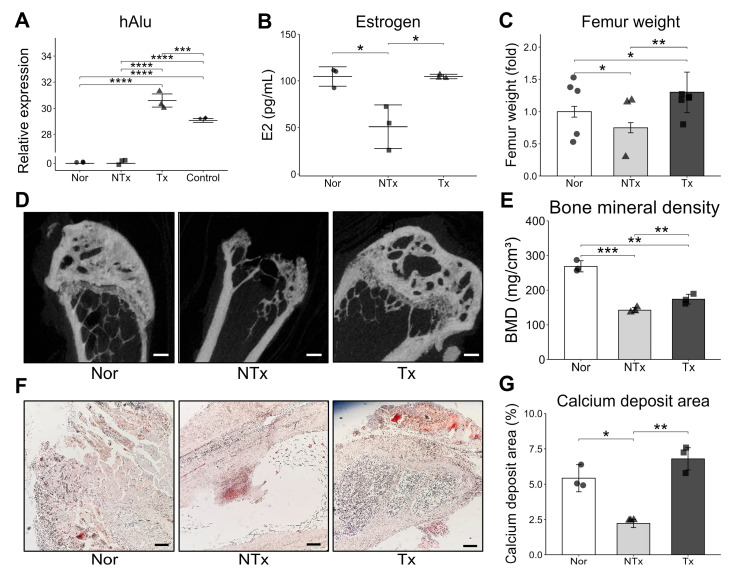
PD-MSC engraftment restores osteoporotic femoral structure in OVX mice. (**A**) Expression of human Alu sequences analyzed by qRT-PCR. (**B**) Estrogen level analyzed by ELISA in mice serum. (**C**) Weight of mice femur. (**D**) Bone structure analyzed by Micro CT. (**E**) Bone mineral density was quantified by imageJ. (**F**,**G**) von Kossa staining performed by sectioned bone tissue and quantified by 3D Histech. Scale bar: 200 μm. Data are shown as mean ± SD. For comparisons involving more than two groups, the Kruskal–Wallis H test was used, followed by the Conover-Iman post hoc test for pairwise comparisons when significant (*p* < 0.05). The Benjamini–Hochberg correction adjusted for multiple testing. A *p*-value < 0.05 was considered significant, * *p* < 0.05; ** *p* < 0.01; *** *p* < 0.001; **** *p* < 0.0001.

To verify the engraftment capability of the cells in vivo, the mRNA expression of the human-specific Alu sequence (hAlu) was examined to quantitatively evaluate the presence of engrafted human cells (Figure 2A). The femur subjected to ovariectomy (OVX) showed notably high expression of hAlu in the Tx group. In contrast, the NTx group did not exhibit significant hAlu expression. Overall, the observed levels of hAlu expression strongly indicate the successful engraftment of PD-MSCs in the femur, a long bone, in vivo, despite being transplanted via tail vein injection.

In a mouse model designed to simulate estrogen deficiency-induced bone loss through OVX, the NTx group displayed a significant decrease in serum estrogen levels, whereas transplantation of PD-MSCs resulted in an increase in estrogen levels (Figure 2B). The outcomes further revealed that conventional markers of osteoporosis, including reduced femur weight, volume, and bone mineral density, significantly improved in the Tx group compared to the NTx group (Figure 2C–E). Moreover, calcium deposits in the Tx group showed substantial recovery (Figure 2F,G). Therefore, these findings emphasize that the transplantation of PD-MSCs effectively alleviates osteoporosis in the femur of OVX mice.

### 2.3. PD-MSCs Exhibited a Bone-Forming Effect in OVX Mice by Restoring the Homeostatic Balance Between Osteoclasts and Osteoblasts

**Figure 3:**
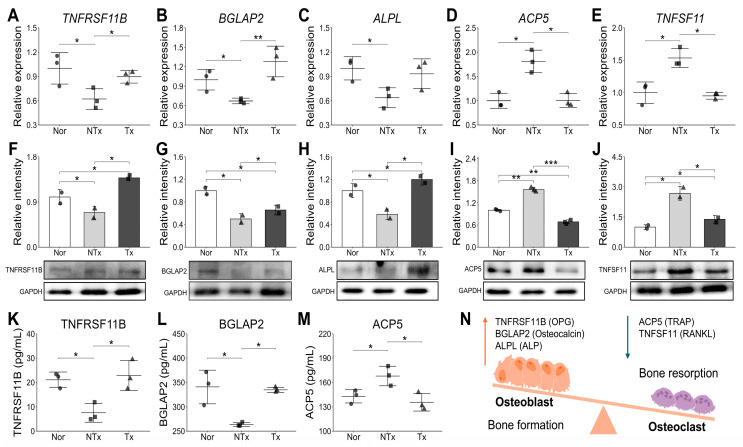
Marker expression patterns of osteoblast and osteoclast in OVX mice and PD-MSCs transplantated mice. The gene expression was analyzed by qRT-PCR for *TNFRSF11B* (**A**), *BGLAP2* (**B**), *ALPL* (**C**), *ACP5* (**D**), and *TNFSF11* (**E**). The protein expression was analyzed by Western blot for TNFRSF11B (**F**), BGLAP2 (**G**), ALPL (**H**), ACP5 (**I**), and TNFSF11 (**J**). The protein levels in serum were analyzed by ELISA for TNFRSF11B (**K**), BGLAP2 (**L**), and ACP5 (**M**). (**N**) The scheme of bone formation balance. Data are shown as mean ± SD. For comparisons involving more than two groups, the Kruskal–Wallis H test was used, followed by the Conover-Iman post hoc test for pairwise comparisons when significant (*p* < 0.05). The Benjamini–Hochberg correction adjusted for multiple testing. A *p*-value < 0.05 was considered significant, * *p* < 0.05; ** *p* < 0.01; *** *p* < 0.001.

The intricate regulation of bone remodeling involves a dynamic interplay between bone-forming osteoblasts and bone-resorbing osteoclasts. Osteoblasts and osteoclasts communicate to modulate cellular behavior, survival, and differentiation, either through direct cell-to-cell contact or by secreting proteins. To investigate the impact of PD-MSCs on the homeostasis between osteoblasts and osteoclasts, we evaluated specific markers for each cell type. Osteoblast markers, including TNFRSF11B, BGLAP2, and ALPL, associated with bone formation, exhibited a significant decrease in the NTx group at both mRNA (Figure 3A–C) and protein levels (Figure 3F–H), while an increase was observed in the Tx group. Conversely, osteoclast-specific markers ACP5 and TNFSF11 (receptor activator of nuclear factor kappa-Β ligand, RANKL) demonstrated increased patterns in the NTx group but decreased in the Tx group at both mRNA (Figure 3D,E) and protein levels (Figure 3I,J). The levels of TNFRSF11B (OPG) and BGLAP2 (Osteocalcin) in serum, indicative of bone formation, were elevated following the transplantation of PD-MSCs (Figure 3K,L). In contrast, the expression levels of ACP5 (TRAP) in serum, representing bone resorption, decreased in the Tx group (Figure 3M). These collective findings suggest that PD-MSCs treatment can balance bone formation by enhancing osteoblast function and diminishing osteoclast function, thereby providing therapeutic effects for osteoporosis (Figure 3N).

### 2.4. PD-MSC Treatment Reduced Inflammatory Cytokines While Increasing Anti-Inflammatory Factors

**Figure 4:**
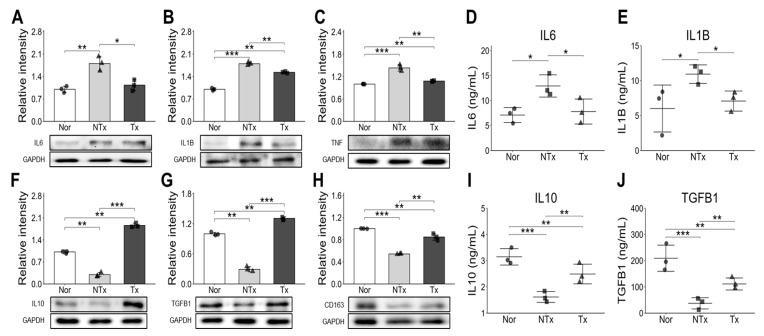
Pro-inflammatory and anti-inflammatory cytokine expression in OVX mice and PD-MSC transplanted mice. Pro-inflammatory cytokine (**A**) IL6, (**B**) IL1B, (**C**) TNF were analyzed by Western blot. (**D**) IL6, (**E**) IL1B level analyzed by ELISA in mice serum. Anti-inflammatory cytokine (**F**) IL10, (**G**) TGFB1, (**H**) CD163 were analyzed by Western blot. (**I**) IL10, (**J**) TGFB1 level analyzed by ELISA in mice serum. Data are shown as mean ± SD. For comparisons involving more than two groups, the Kruskal–Wallis H test was used, followed by the Conover-Iman post hoc test for pairwise comparisons when significant (*p* < 0.05). The Benjamini–Hochberg correction adjusted for multiple testing. A *p*-value < 0.05 was considered significant, * *p* < 0.05; ** *p* < 0.01; *** *p* < 0.001.

We conducted a protein analysis and blood chemistry analysis to verify the inflammatory response after PD-MSC injection. The results revealed a significant decrease in pro-inflammatory factors such as IL6, IL1B and TNF in the Tx group compared to the NTx group, while an increase was observed in the NTx group compared to the normal group (Figure 4A–E). Conversely, the anti-inflammatory factors IL10, TGFB1, and CD163 exhibited a significant increase in the Tx group compared to the NTx group (Figure 4F–J). These findings suggest that the engrafted PD-MSCs at the injured bone tissue site, through transplantation, effectively alleviates the inflammatory response in the OVX mouse model.

### 2.5. Transplantation of PD-MSCs Enhanced Bone Morphogenic Protein (BMP) Signaling Pathway for Bone Formation

**Figure 5:**
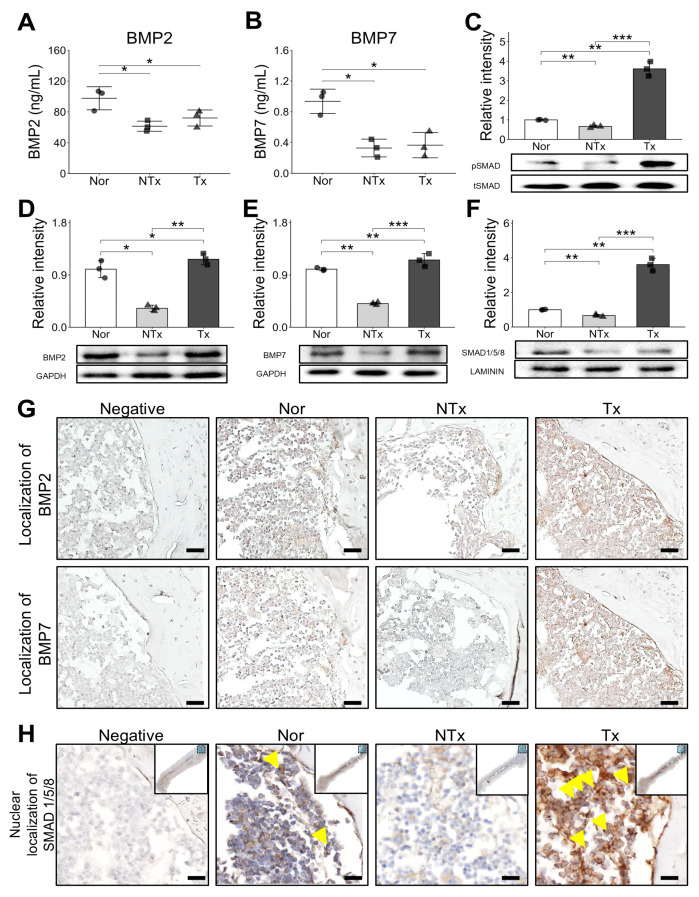
Bone morphogenic protein (BMP) and SMAD expressions in OVX mice and PD-MSC transplanted mice. (**A**) Bone morphogenic protein 2 (BMP2) and (**B**) Bone morphogenic protein 7 (BMP7) level analyzed by ELISA in mice serum. (**C**) Phosphorylated SMAD1/5/8 protein expression was analyzed by Western blot. (**D**) Bone morphogenic protein 2 (BMP2) and (**E**) Bone morphogenic protein 7 (BMP7) protein expressions were analyzed by Western blot. (**F**) SMAD protein expression in nucleus was analyzed by Western blot. (**G**) Representative images showing Bone morphogenic protein 2 (BMP2) and Bone morphogenic protein 7 (BMP7) expression in trabecular bone regions. Positive staining was primarily observed in osteoblasts and osteocytes lining the trabecular surface. Scale bar: 100 μm. (**H**) High-magnification images showing nuclear localization of SMAD1/5/8 (yellow arrows) in osteoblasts and osteocytes. PD-MSC transplantation (Tx) markedly restored Bone morphogenic protein 2,7 (BMP2,7) expression and SMAD1/5/8 activation compared with the OVX untreated group (NTx). Scale bar: 50 μm. Data are shown as mean ± SD. For comparisons involving more than two groups, the Kruskal–Wallis H test was used, followed by the Conover-Iman post hoc test for pairwise comparisons when significant (*p* < 0.05). The Benjamini–Hochberg correction adjusted for multiple testing. A *p*-value < 0.05 was considered significant, * *p* < 0.05; ** *p* < 0.01; *** *p* < 0.001.

The principal signaling pathways governing the bone regenerative process include Bone morphogenic protein (BMP) signaling. Bone homeostasis relies on the balanced function of osteoblasts and osteoclasts, and key regulators in this process have been extensively studied [20]. Bone morphogenic protein (BMP), members of the transforming growth factor-beta (TGF-β) superfamily, play a crucial role in various cell regulatory processes, including osteogenic differentiation and the regulation of bone formation through SMADs [21]. To explore the effects of PD-MSC transplantation on Bone morphogenic protein (BMP) signaling, we assessed the levels of Bone morphogenic protein 2 (BMP2) and Bone morphogenic protein 7 (BMP7) proteins, along with SMAD1/5/8. In OVX mice, Bone morphogenic protein 2 (BMP2) and Bone morphogenic protein 7 (BMP7) levels in serum were downregulated, but PD-MSCs treatment significantly increased these Bone morphogenic protein (BMP) signaling components (Figure 5A,B,D,E). Phosphorylated SMAD1/5/8, as activated R-SMAD forms, levels decreased in the NTx group, but PD-MSC treatment significantly increased phosphorylated SMAD1/5/8 levels (Figure 5C). Nuclear localization of activated SMADs was also analyzed by Western blot after nuclear fractionation. The expression of SMAD levels in nucleus decreased in the NTx group but increased in the Tx group (Figure 5F). Immunohistochemistry of sectioned bone tissues demonstrated increased levels of Bone morphogenic protein 2 (BMP2) and Bone morphogenic protein 7 (BMP7) in the bone tissue of the Tx group (Figure 5G). Notably, the nuclear translocation of SMADs increased in the Tx group (Figure 5H). These findings indicate that PD-MSC acts to prevent osteoporosis by enhancing signaling pathways associated with BMPs/SMADs.

## 3. Discussion

Postmenopausal osteoporosis, which arises from estrogen deficiency, lead to reduced bone mass and increased fracture risk [22]. While traditional treatments like estrogen replacement and bisphosphonates are effective, they carry significant risks [23]. However, PD-MSC therapy offers a promising alternative with fewer side effects [17]. Previous study has shown that PD-MSCs contribute to bone repair by secreting bioactive molecules, such as Bone morphogenic protein (BMP), TGF-β, and VEGF, which support osteogenesis [24]. According to research by Janet L. Crane, Xu Cao, and colleagues, mesenchymal stem cells particularly help restore Bone morphogenic protein (BMP)-SMAD signaling, crucial for bone metabolism [25].

Our study demonstrated that OVX mouse model showed decreased estrogen, bone mineral density, femur weight, and calcium deposition but were increased after PD-MSCs transplantation, which contributed to bone loss. Also, we found that PD-MSCs enhance osteogenic markers and reduce bone resorption makers, suggesting their potential to prevent bone density loss.

Previous studies on stem cell therapy for osteoporosis have primarily focused on the differentiation of stem cells into osteoblasts to promote bone formation. This approach aimed to utilize the regenerative potential of stem cell differentiation to restore damaged bone tissue [26]. However, our research presents a different perspective by suggesting that osteoporosis can potentially be overcome through various bioactive factors secreted by stem cells [27]. These factors, which possess regenerative, anti-inflammatory, and antioxidant properties, offer therapeutic benefits without relying solely on the differentiation of stem cells. This approach emphasizes the therapeutic effects of secreted factors from stem cells, providing an alternative to traditional differentiation-based methods.

In this study, we confirmed that estrogen deficiency occurs in the OVX model, which is commonly used to mimic postmenopausal osteoporosis [28]. Notably, following stem cell transplantation, we observed significant increases in estrogen levels, femur weight, bone mineral density (BMD), and calcium deposition intensity. These improvements suggest that stem cell therapy may positively impact bone health and mitigate the effects of estrogen deficiency, contributing to the restoration of bone structure and strength. Also, our study found that the PD-MSCs transplantation activate bone formation markers and decrease bone resorption markers (Figure 3), indicating its potential to rebalance bone remodeling. This process is closely regulated by the interplay between key molecular factors, including TNFRSF11B, BGLAP2, ALPL, ACP5, and TNFSF11, which play essential roles in maintaining bone homeostasis [29]. This study revealed distinct differences in bone formation and resorption markers between the NTx and Tx groups. Bone formation-related markers such as TNFRSF11B (Osteoprotegerin/OPG), which acts as a decoy receptor inhibiting osteoclast differentiation and bone resorption, and BGLAP2 (Osteocalcin) and ALPL (Alkaline Phosphatase), which play essential roles in osteoblast activity and mineralization [30], exhibited a significant decrease in both mRNA and protein levels in the NTx group, while showing an increasing trend in the Tx group. This suggests that bone formation capacity was inhibited in the NTx group, whereas it was promoted or restored to normal levels in the Tx group. Conversely, osteoclast-specific markers ACP5 (TRAP), an indicator of osteoclast activity during bone resorption, and TNFSF11 (RANKL), a key factor promoting osteoclast activation and bone breakdown [31], showed increased patterns in both mRNA and protein levels in the NTx group but decreased in the Tx group. These results clearly indicate that the NTx group was in an imbalanced state with dominant bone resorption, whereas the Tx group strongly suggests that the transplantation worked towards improving bone homeostasis by inhibiting osteoclast activity and reducing bone resorption.

Through these results, we understanded that the possibility of estrogen deficiency disrupts bone remodeling by shifting the balance between bone formation and resorption, as it normally inhibits osteoclast activity and promotes osteoblast function.

Additionally, estrogen deficiency triggers the release of inflammatory cytokines such as IL-6, IL-1β, which further stimulate osteoclast activity and accelerate bone breakdown [32]. Our study found elevated levels of pro-inflammatory cytokines (IL-6, IL-1β, TNF-α) in the OVX group, which decreased with PD-MSC transplantation. However, anti-inflammatory cytokines (IL-10, TGF-β, CD163) increased in the PD-MSC group (Figure 4). Notably, as shown Figure 4C, TNF-α inhibits Bone morphogenic protein (BMP)-induced osteoblastic differentiation by suppressing BMP-SMAD signaling [33].

Cell-based regenerative medicine shows great promise for treating osteoporosis by modulating bone resorption, reducing fracture risk, and enhancing mineral density. The repair of bone tissue involves local signals from cytokines and growth factors that promote the migration, differentiation, and proliferation of osteoprogenitor cells, as well as revascularization and extracellular matrix production [26,34]. Mesenchymal stem cells (MSCs) support bone regeneration by secreting bioactive molecules like Bone morphogenic protein (BMP), IGF-1, TGF-β, VEGF, angiogenin, HGF, and IL-6 [35,36].

In our study, we observed an increase in Bone morphogenic protein (BMP) and SMAD expression following stem cell transplantation, as confirmed through ELISA, Western blot, and staining (Figure 5). Additionally, SMAD expressions were evaluated in the nuclear fraction of samples, where we noted a consistent pattern. Immunohistochemistry (IHC) staining further revealed the presence of SMAD in the nucleus, confirming its nuclear localization and supporting the role of SMAD in mediating signaling pathways within the cell nucleus. We also observed increased nuclear SMAD levels in the PD-MSC group, suggesting that PD-MSCs may help reverse impaired BMP/SMAD signaling involved in bone metabolism.

Supporting this idea, we observed that Bone morphogenic protein (BMP) signaling initiated by factors secreted from stem cells plays a crucial role in activating SMAD proteins, which then translocate into the nucleus. Once inside the nucleus, SMAD proteins act as transcription factors, regulating gene expression that supports bone metabolism and promotes anti-inflammatory responses. The BMP/SMAD signaling pathway is essential for maintaining bone homeostasis [37,38]. Specifically, Bone morphogenic protein (BMP) triggers SMAD phosphorylation, enabling SMAD proteins to enter the nucleus where they influence the expression of genes associated with osteogenesis and anti-inflammatory processes [39]. Increased nuclear localization of SMAD following Bone morphogenic protein (BMP) activation has been linked to enhanced osteoblast activation and bone matrix formation, which is critical in counteracting bone loss due to osteoporosis. This pathway not only promotes bone formation but also supports anti-inflammatory effects by regulating the expression of cytokines and other mediators involved in immune responses.

Our findings underscore the therapeutic potential of stem cell-derived Bone morphogenic protein (BMP) signaling in promoting bone regeneration and controlling inflammation. By facilitating SMAD activation, stem cell therapy offers a multifaceted approach to managing osteoporosis, improving bone density, and reducing inflammation through molecular mechanisms that enhance both bone metabolism and anti-inflammatory pathways. While our findings provide significant insights into the therapeutic effects of PD-MSCs on osteoporosis, further research is needed to build upon these results and address certain limitations. Firstly, larger and more diverse study populations are required to confirm and generalize our findings. Secondly, a deeper investigation into the underlying molecular and cellular mechanisms is necessary to elucidate the precise pathways involved, particularly by further exploring the BMP/SMAD signaling pathway in vitro.

**Figure 6:**
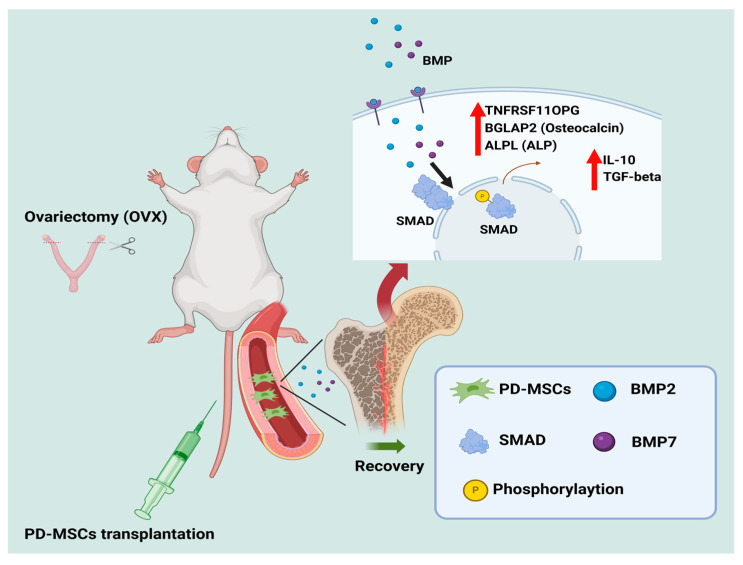
An imbalance in a dynamic equilibrium of bone remodeling leads to osteoporosis being recovered by PD-MSCs injection.

Overall, this study provides compelling evidence that PD-MSC transplantation can regulate osteogenesis and modulate inflammation through its anti-inflammatory effects, in conjunction with BMP/SMAD signaling (Figure 6). These findings offer new insights into the therapeutic potential of PD-MSCs for osteoporosis treatment, demonstrating their ability to support bone homeostasis through anti-osteoclast and anti-inflammatory paracrine effects.

## 4. Materials and Methods

### 4.1. Cell Culture

Placentas were collected from women who were free of any medical, obstetrical, or surgical complications and who delivered at term (38 ± 2 gestational weeks). PD-MSCs were isolated from human placental chorionic plates and approved by the Institutional Review Board of CHA General Hospital, Seoul, Republic of Korea (IRB 07-18). PD-MSCs were isolated from chorionic plates of normal-term placentas. Briefly, PD-MSCs were cultured in alpha-minimum essential medium (α-MEM; Hyclone, GE healthcare life sciences, Seoul, Republic of Korea) supplemented with 10% fetal bovine serum (FBS; Gibco-BRL, Rockville, MD, USA), 1% penicillin/streptomycin (Pen-Strep; Gibco-BRL), 25 μg/mL human fibroblast growth factor 4 (hFGF-4; Peprotech Inc., Rocky Hill, NJ, USA), and 1 μg/mL heparin (Sigma-Aldrich, St. Louis, MO, USA) at 37 °C in an incubator with a humidified atmosphere of 5% CO_2_.

### 4.2. Animal Model and Transplantation of PD-MSCs

Seven-week-old female C57BL/6 mice (Orient Bio Inc., Seongnam, Republic of Korea; n ≥ 3 per group) were housed in a temperature- and humidity-controlled facility (22 ± 2 °C, 50 ± 10% humidity, 12 h light/dark cycle) with free access to food and water and acclimatized for one week before experimentation. After acclimatization, all animals were randomly allocated into two groups: a non-transplantation (NTx) group consisting of ovariectomized (OVX) mice, and a transplantation (Tx) group consisting of OVX mice that injected PD-MSC transplantation via tail-vein injection. Ovariectomy was performed under isoflurane anesthesia by making a small dorsal incision, ligating, and removing both ovaries after sterilization with 70% ethanol, followed by povidone-iodine disinfection. All OVX mice were maintained for four weeks post-surgery to establish the osteoporotic condition. Thereafter, mice in the Tx group were intravenously administered PD-MSCs (5 × 10^4^ cells in 100 µL PBS) through the tail vein, whereas NTx mice injected an equal volume of PBS. Six weeks after transplantation, blood samples were collected for hormone analysis, and mice were sacrificed for bone tissue harvesting. All experimental procedures were approved by the Institutional Animal Care and Use Committee (IACUC) of CHA University (IACUC 220044) and conducted in accordance with institutional and national animal welfare regulations. Furthermore, all in vivo experiments were performed in compliance with the ARRIVE 2.0 (Animal Research: Reporting of In Vivo Experiments) guidelines [40] to ensure methodological transparency, reproducibility, and ethical integrity.

### 4.3. Flow Cytometry Analysis

To phenotype cell-surface antigens, PD-MSCs were incubated with each of the following monoclonal antibodies: anti-CD34-PE, anti-CD90-PE, anti-HLA-ABC-FITC, anti-HLA-DR-FITC (BD Bioscience, San Jose, CA, USA), anti-CD45-FITC (BioLegend, San Diego, CA, USA), anti-CD105-FITC (R&D Systems, Abingdon, UK). The phenotype of each cell line was analyzed using a FACSCalibur flow cytometer (Becton Dickinson, San Jose, CA, USA).

### 4.4. Reverse Transcriptase-Polymerase Chain Reaction (RT-PCR) of RNA from PD-MSCs

Total RNA was isolated from PD-MSCs using a TRIzol reagent (Gibco BRL, Rockville, MD, USA). cDNA synthesis was performed according to the protocol of the DiaStar™ OneStep RT-PCR Series (SolGent Co., Ltd., Daejeon, Republic of Korea). The PCR conditions were as follows: 95 °C/4 min (initial melting); 35 cycles of amplification at 95 °C/30 s, 50–57 °C/30 s, 72 °C/30 s; and a final extension at 72 °C/3 min. To analyze stemness and differentiation markers in PD-MSCs, the PCR products were electrophoresed and imaged on 2% agarose gels by staining with Gel Red (Sigma-Aldrich). The primer sequences are included below (Table 1).

### 4.5. Multilineage Differentiation of PD-MSCs

To induce adipogenic and osteogenic differentiation, PD-MSCs were induced in medium from StemPro adipogenesis and osteogenesis differentiation kits for MSCs (Gibco, Thermo Fisher Scientific, Waltham, MA, USA). We changed the medium every other day. After approximately 21 days, cells transfected with each system were fixed with 4% PFA, oil red O (Sigma-Aldrich) staining of lipid was carried out to visualize lipid vesicles, and von Kossa staining was performed with 5% silver nitrate (Sigma-Aldrich) under light to evaluate the accumulation of calcium deposits. To differentiate into chondrogenic lineage, PD-MSCs using both systems were induced in medium from MesenCult-ACF chondrogenic differentiation kit for MSCs (Stem Cell Technologies, Vancouver, BC, Canada) for approximately 21 days. Each sample was fixed with 4% PFA and stained with 1% alcian blue solution (pH 2.5; Sigma-Aldrich) to identify chondrocytes.

### 4.6. SA-β-Gal Staining

The senescence activity of each cell type (passage no. 3) was detected using an SA-β-gal kit (Cell Signaling, Danvers, MA, USA) according to the manufacturer’s instructions. Cells were fixed for 10 min in a 1× fixative solution at room temperature and then incubated overnight at 37 °C with a 1× SA-β-gal staining solution (pH 6.0). The percentage of SA-β-gal-positive cells among each cell type was analyzed by the program ImageJ (v1.54g, NIH).

### 4.7. Micro CT Analysis

To examine the bone mineral density in OVX model, the rabbits were sacrificed after 6 weeks, and their tissue were harvested. After decalcification, the samples were processed and scanned using Micro-CT system (MicroCT 45, SCANCO Medical AG, Wangen-Brüttisellen, Switzerland) at a voltage of 90 kV, and electric current of 88 μA, and exposure time of 500 ms with X-ray setting. The system magnification was set to 3.70 mm and 500 slices were taken at 18 μm/voxel. Subsequently, reconstructed three-dimensional (3D) tomograms were analyzed with Scano Medical Software (version 1.1) to calculate the bone volume (BV). The percentage of BV to the total tissue volume (TV) within the volume of interest (VOI) was analyzed and expressed as the mean ± standard deviation.

### 4.8. Histological Analysis

Isolated femora were fixed in 10% neutral-buffered formalin transferred to 70% ethanol, and decalcified in 9% formic acid. After tissue processing, the specimens were embedded in paraffin. Sections (5 μm) were cut sagittally along the femoral shaft axis and collected on glass slides, deparaffinized, and subjected to hematoxylin and eosin (H&E) staining using standard protocols. After mounting with coverslips, the specimens were viewed and analyzed under a light microscope. Bone sections were stained with Von Kossa stain to determine calcium deposition, and calcium were quantified using Image J 1.54 software.

### 4.9. Quantitative Real-Time Polymerase Chain Reaction (qRT-PCR) Analysis of RNA from Bone Tissue

Bone tissues from mice were extracted and rapidly frozen. Total RNA was isolated after the tissues had been homogenized. Each sample group received 0.2 mL of chloroform and 1 mL of TRIzol reagent (Invitrogen, Carlsbad, CA, USA) before centrifugation to separate the supernatant. The particles were obtained by washing the separated supernatant with isopropyl alcohol/ethyl alcohol and discarding it. The pellet was subsequently dissolved in water treated with DEPC (Invitrogen) at 60 °C. The concentration of total RNA was determined using a Nanodrop spectrophotometer (Thermo Fisher Scientific, Waltham, MA, USA). Superscript III reverse transcriptase (Invitrogen) was used to convert the whole RNA into cDNA. The following are the PCR requirements for cDNA synthesis: 5 min at 65 °C, 1 min at 4 °C, 60 min at 50 °C, and 15 min at 72 °C. qRT-PCR was performed using FS Universal SYBR Green Master ROX and cDNA (Roche, Basel, Switzerland). Subsequently, the cDNA was amplified by PCR under the following conditions: 5 s at 95 °C, followed by 40 cycles of 5 s at 95 °C and 30 s at 60 °C. Each sample was examined in triplicate, with rat GAPDH as the internal control for standardization. Table 2 lists the qRT-PCR primer sequences.

### 4.10. Nuclear Fraction

Nuclear and cytoplasmic protein fractionation was performed using the NE-PER Nuclear and Cytoplasmic Extraction Reagents (Thermo Fisher Scientific) following the manufacturer’s protocol with slight modifications to optimize tissue sample processing. Bone tissue samples (50–100 mg) were collected and immediately washed with cold phosphate-buffered saline (PBS) to remove excess blood and other contaminants. The samples were then homogenized in 200 µL of ice-cold Cytoplasmic Extraction Reagent I (CER I), supplemented with protease inhibitors (Thermo Fisher Scientific). Following homogenization, the tissue lysates were transferred to microcentrifuge tubes and incubated on ice for 10 min. Cytoplasmic Extraction Reagent II (CER II, 11 µL) was then added, and the mixture was briefly vortexed for 5 s. After an additional 1 min incubation on ice, the samples were centrifuged at 16,000× *g* for 5 min at 4 °C. The supernatant, containing the cytoplasmic fraction. The remaining pellet was resuspended in 100 µL of ice-cold Nuclear Extraction Reagent (NER), supplemented with protease inhibitors, and vortexed for 15 s. The samples were incubated on ice for 40 min, with intermittent vortexing every 10 min to enhance nuclear protein solubilization. Following incubation, the samples were centrifuged at 16,000× *g* for 10 min at 4 °C, and the supernatant containing nuclear proteins were carefully collected and stored on ice or at −80 °C for long-term storage.

### 4.11. Western Blot Analysis

Bone tissues from each group were homogenized and lysed on ice using RIPA buffer (Sigma-Aldrich, USA), which contains a phosphatase inhibitor cocktail and a protease inhibitor cocktail (genDEPOT, Altair, TX, USA). Using a bicinchoninic acid test (BCA) protein assay kit (Thermo Fisher Scientific), the protein concentration from individual rats was measured and standardized to be the same. Protein extracts of equal concentration were separated by electrophoresis on 9% sodium dodecyl sulfate-polyacrylamide gels (SDS–PAGE). Using a turbo transfer system, the separated proteins were transferred onto a polyvinylidene difluoride (PVDF) membrane (Bio-Rad Laboratories, Hercules, CA, USA). The membranes were blocked in blocking solution (5% BSA) for 1 h at room temperature. The membranes were then exposed to the primary antibody in 2% BSA overnight at 4 °C. The following antibodies were mixed with 2% BSA and incubated at 4 °C. After incubation, the membranes were washed with 1× Tris-buffered saline-Tween 20 (TBS-T) and incubated with secondary antibody (1:10,000) at room temperature for 1 h according to the manufacturer’s instructions. Membranes were washed before exposure to the Clarity Western ECL kit (Bio-Rad Laboratories) for 5 min at room temperature. The Chemi Doc XRS+ imaging technology was used to identify the protein bands (Bio-Rad Laboratories). ImageJ software (Wayne Rasband, Bethesda, MD, USA) was used to analyze the bands. The fold change value of intensity is the comparative value of gene expression.

### 4.12. Enzyme-Linked Immunosorbent Assay (ELISA)

All blood samples were collected from the aorta in mice of NTx and Tx groups. The serum samples were separated from whole blood by using a blood collection tube (vacutainer; BD Biosciences, San Jose, CA, USA) at 1300 RCF for 15 min. All blood serum was stored at −80 °C and the estrogen, TRAP, OPG, Osteocalcin activity in serum were analyzed by ELISA kits, following the manufacturer’s instructions. In brief, an equal volume of sample was added to the specific antibody-coated plates. Next, the specific horseradish peroxidase (HRP)-conjugates were added to each well and incubated at 37 °C. After the substrates had been added and incubated in the dark for substrate development, the antibody activity was analyzed by using a microplate reader (BioTek, Winooski, VT, USA). All samples were tested three times, and the results are presented as the relative value.

### 4.13. Statistical Analysis

Data are presented as mean ± standard deviation (SD). To compare two independent groups, we employed the non-parametric Mann–Whitney U test (also referred to as the Wilcoxon rank; -sum test). This test was chosen due to its suitability for non-normally distributed data and its reliance on rank-based comparisons, making it appropriate for both ordinal and continuous data that do not meet the assumption of normality. A *p*-value of less than 0.05 was considered statistically significant. For comparisons involving more than two groups, the Kruskal–Wallis H test, a non-parametric alternative to one-way ANOVA, was applied to assess whether there were statistically significant differences between group medians. This test is suitable for non-parametric data as it does not assume normal distribution. The analysis was conducted using the kruskal.test function in R (v4.4.0). If the Kruskal–Wallis test showed a statistically significant result (*p* < 0.05), pairwise comparisons between groups were conducted using the Conover-Iman post hoc test. This test, appropriate for non-parametric data, allows for multiple pairwise comparisons following a significant Kruskal–Wallis test [41]. Post hoc comparisons were performed using the ConoverTest function from the DescTools (v0.99.54) package [42]. To account for multiple testing and reduce the risk of Type I errors, the Benjamini–Hochberg correction was applied to adjust the *p*-values. This correction controls the false discovery rate (FDR), minimizing the risk of false positives in multiple hypothesis testing. Adjusted *p*-values were computed using the ConoverTest function in DescTools (v0.99.54), applying the “BH” method. All statistical analyses were performed using R (v4.4.0) and RStudio (v2024.04.0+735) [43]. A *p*-value of less than 0.05 was considered statistically significant for all tests.

## Figures and Tables

**Table 1:** Primers for RT-PCR of RNA from PD-MSCs.

Gene	Primer	Tm (°C)	Size (bp)
*POU5F1* *(OCT4)*	F: 5′-AGTGAGAGGCAACCTGGAGA-3′	52	273
	R: 5′-GTGAAGTGAGGGCTCCCATA-3′		
*NANOG*	F: 5′-TTCTTGACTGGGACCTTGTC-3′	52	260
	R: 5′-GCTTGCCTTGCTTTGAAGCA-3′		
*SOX2*	F: 5′-GGGCAGCGTGTACTTATCCT-3′	52	200
	R: 5′-AGAACCCCAAGATGCACAAC-3′		
*HLA-G*	F: 5′-GCGGCTACTACAACCAGAGC-3′	58	550
	R: 5′-GCACATGGCACGTGTATCTC-3′		
*NEFL*	F: 5′-GAGTGAAATGGCACGATACCTA-3′	58	500
	R: 5′-TTTCCTCTCCTTCTTCTTCACCTTC-3′		
*ACTC1*	F: 5′-GGAGTTATGGTGGGTATGGGTC-3′	58	500
	R: 5′-AGTGGTGACAAAGGAGTAGCCA-3′		
*AFP*	F: 5′-AGCTTGGTGGATGAAAC-3′	50	200
	R: 5′-TCCAACAGGCCTGAGAAATC-3′		
*ACTB* *(β-actin)*	F: 5′-TCCTTCTGCATCCTGTCAGCA-3′	58	300
	R: 5′-CAGGAGATGGCCACTGCCGCA-3′		

**Table 2:** Primers for qRT-PCR of RNA from bone tissue.

Gene	Primer	Tm (°C)
*hAlu*	F: 5′-GGAGGCTGAGGCAGGAGAA-3′	55
	R: 5′-CGGAGTCTCGCTCTGTCGCCCA-3′	
*TNFRSF11B (OPG)*	F: 5′-GTGTGTCCCTTGCCCTGACTAC-3′	60.8
	R: 5′-GTTTCACGGTCTGCAGTTCCTT-3′	
*ALPL(ALP)*	F: 5′-TGAATCGGAACAACCTGACTGA-3′	59.1
	R: 5′-TTCCACTAGCAAGAAGAAGCCTTT-3′	
*BGLAP2(Osteocalcin)*	F: 5′-AAGCCCAGCGACTCTGAGTCT-3′	60.5
	R: 5′-GCTCCAAGTCCATTGTTGAGGTA-3′	
*ACP5(TRAP)*	F: 5′-CGCCAGAACCGTGCAGA-3′	59.1
	R: 5′-TCAGGCTGCTGGCTGAC-3′	
*TNFSF11(RANKL)*	F: 5′-AACTGGTCGGGCAATTCTGA-3′	59.2
	R: 5′-GGGTTCGACACCTGAATGCT-3′	
*mGAPDH*	F: 5′-GCACCGTCAAGGCTGAGAAC-3′	60
	R: 5′-GTGGTGAAGACGCCAGTGGA-3′	

## Data Availability

All data generated or analyzed during this study are included in this manuscript files.
